# Synthesis of causal and surrogate models by non-equilibrium thermodynamics in biological systems

**DOI:** 10.1038/s41598-024-51426-8

**Published:** 2024-01-10

**Authors:** Kazuhiro Sakurada, Tetsuo Ishikawa

**Affiliations:** 1 Department of Extended Intelligence for Medicine, The Ishii-Ishibashi Laboratory, Keio University School of Medicine, Tokyo, Japan; 2 Open Systems Information Science Team, Advanced Data Science Project, RIKEN Information R&D and Strategy Headquarters, RIKEN, Tokyo, Japan; 3 Medical Data Mathematical Reasoning Team, Advanced Data Science Project, RIKEN Information R&D and Strategy Headquarters, RIKEN, Yokohama, Japan

**Keywords:** Biophysics, Systems biology

## Abstract

We developed a model to represent the time evolution phenomena of life through physics constraints. To do this, we took into account that living organisms are open systems that exchange messages through intracellular communication, intercellular communication and sensory systems, and introduced the concept of a message force field. As a result, we showed that the maximum entropy generation principle is valid in time evolution. Then, in order to explain life phenomena based on this principle, we modelled the living system as a nonlinear oscillator coupled by a message and derived the governing equations. The governing equations consist of two laws: one states that the systems are synchronized when the variation of the natural frequencies between them is small or the coupling strength through the message is sufficiently large, and the other states that the synchronization is broken by the proliferation of biological systems. Next, to simulate the phenomena using data obtained from observations of the temporal evolution of life, we developed an inference model that combines physics constraints and a discrete surrogate model using category theory, and simulated the phenomenon of early embryogenesis using this inference model. The results show that symmetry creation and breaking based on message force fields can be widely used to model life phenomena.

## Introduction

The challenges facing humanity are related to complex systems such as human, social and ecological systems. A fundamental role of science is to predict the behavior of complex systems and to prevent problems from occurring. In biology, individual phenomena have been explained by causal mechanisms rather than by the governing equations of physics^1–3^. On the other hand, advances in machine learning techniques and computing power have made it possible to construct surrogate models that mimic the temporal evolution of biological phenomena from large amounts of data^4^. However, most of the surrogate models used in biomedical sciences are black box models, which means that the reproducibility and reliability of the models are affected by the bias of the data used for training^5^. To overcome this problem, it is necessary to incorporate physics-based constraints into the surrogate model in addition to the mechanistic constraints that have been identified in previous empirical studies in biology and medicine.

Boltzmann adopted the model of statistical mechanics, in which the system is composed of a vast number of moving particles, and explained the second law of thermodynamics in the form that the system moves toward a disordered state described by the largest number of microscopic states with the highest possible probability^6^. In the modern synthesis established in the 1940s, this Boltzmann interpretation and the spontaneous creation of order observed in biological evolution were considered inconsistent, and mechanistic rather than physics-based constraints were adopted as a way to explain biological phenomena^7^. In contrast, Schrödinger^8^ and Bertalanffy^9^ argued that the transformation from disorder to order, as observed in biological systems, does not violate the second law of thermodynamics as long as such systems produce enough entropy to compensate for their own internal entropy reduction. Prigogine showed that entropy production in a system is minimized in a nonequilibrium steady state with constant total thermodynamic quantities^10^.

Organisms are open systems driven far from thermodynamic equilibrium. Jarzynski extended Clausius' inequality to conditions far from equilibrium^11^. Crooks characterized the probability of breaking the second law by the fluctuation theorem^12^. England presented thermodynamic constraints on the behavior of systems far from equilibrium. This is the principle that when energy is poured into a system from outside in the presence of thermal fluctuations, most of the changes in the system are random, but irreversible changes occur when the system more efficiently absorbs and dissipates free energy. England called this principle dissipative adaptation^13^. These three principles provided a unified explanation for the nonequilibrium phenomena expressed in the particle model.

However, as long as we use models of particles without interactions, the physics-based constraints cannot be fully incorporated into the description of life phenomena. This is because biological behavior is signal-based. Biological systems consist of numerous hierarchies, from cells to ecosystems, and a high degree of coordination is observed^3,14^. Such coordination is possible because of the diverse messages exchanged at all levels of the biological hierarchy^14^. Messages generated by biological systems include chemical, visual, and auditory information. Messages consisting of chemical substances include intracellular signaling molecules, intercellular signaling molecules, and molecules exchanged between individual organisms. The colors and shapes of living organisms become visual information through light. The physical vibrations emitted by living organisms become auditory information through sound. Until now, messages exchanged between biological systems have been explained by mechanistic constraints on how they are transmitted and how that information is processed.

When spontaneous motion occurs in nature, it manifests itself as periodic motion^15^. Oscillators have the property of synchronizing when they are connected by weak interactions. Messages exchanged in biological systems induce synchronization between systems by changing the state of the system receiving them. The steady state generated by synchronization between oscillators is broken by the disappearance of signaling. We show in this paper that the temporal evolution of biological systems can be described by physics-based constraints by positing oscillators as the basic dynamic unit and modeling the different patterns of self-organization as caused by synchronization of systems by messages or desynchronization by fluctuations.

Biological systems can be represented by three different models. In biology and medicine, living organisms are represented by types and functions^1^. In the surrogate model, the state of the system under consideration is represented by a vector at a single point in space as a set of variables^16^. The temporal evolution of the state is represented by the trajectory of a point in space that moves with the evolution of time^17^. The space that is a collection of states is called a phase space or state space^18^. In dynamics, trajectories on phase space are considered to be attracted to a certain collection of states. Such a region is called an attractor. The behavior of the attractor states governs the steady-state characteristics of the system. We show in this paper that category theory allows us to synthesize three models of biological systems described from different perspectives.

## Results

### Interaction through messages in biological systems

Organisms are self-organizing open systems. Self-organization is a spatio-temporal process of acquiring structure and function that takes place without specific external commands. Such self-organization occurs in systems that are far from thermal equilibrium. The state of a biological system is changed by the interactions of the subsystems that comprise it. On the other hand, the behavior of the subsystem changes with the state of the overall system. Thus, the hierarchies in close proximity interfere with each other in a nonlinear fashion.

An organism is a multiscale system consisting of intracellular organelles, cells, organs, individuals, and ecosystems. At each hierarchy, systems interact with the outside world by exchanging matter, energy, and messages. Biological systems differ from non-living systems in that they can send messages and receive messages from the outside^14^.

The animal prey receives messages from the animal predator through the five senses and transitions its physical state to fight or flight^19^. The appearance of an organism functions as a message because animals have acquired the ability to see. When plants are preyed upon by insects or large mammals, they produce a message called Volatile Organic Compounds (VOC)^20^. Surrounding plants that receive VOCs will transition their systems to a defensive state. When humans are infected by bacteria or viruses, innate and acquired immune responses occur. In the innate immune response, dendritic cells and macrophages recognize antigens and produce messages called cytokines^21^. Upon receiving the cytokine message, immune cells change to an inflammatory state and prevent the growth of pathogens. In this way, messages are spread throughout the multiple layers of the organism.

In both unicellular and multicellular organisms, messages are received via receptors expressed in the cell. If the message is a chemical, it is received by receptors that selectively identify it^22^. Light messages are received by photoreceptors^23^. Mechanical stimulus such as sound or touch are received by ion channels in the hair cells of the inner ear or Merkel cells in the skin^24^. Upon receiving a message, the cell transmits the message to the chromosomes via intracellular signaling pathways, resulting in changes in gene expression^25^. As a result, the state of the cell changes. In the case of multicellular organisms, changes in the cell receiving the message are transmitted to other cells and tissues. Thereby, multiple messages received simultaneously by the multicellular organism are integrated and the real world is perceived.

The force of a message to change the state of a cell is indicated by the binding constant of the message to its receptor on the cell membrane. If the message is a molecule, the force of the message is indicated by the binding constant to the receptor that selectively binds to the molecule^26^. If the message is light, it is received by photoreceptors. One of the photoreceptors present in the animal eye is rhodopsin. The light message is received by isomerization of the inactive 11-cis retinal covalently bound to rhodopsin to the active all-trans form upon absorption of light^23^. Since the amount of active retinal increases with light intensity, the force of the light message is indicated by the binding constant between the active retinal and the photoreceptor. If the message is a mechanical stimulus, such as sound, pressure, or tension, the message is received when the mechanical stimulus deforms the cell membrane and opens the ion channel^24^. Since the amount of deformed lipid membrane increases with the intensity of the mechanical stimulus, the force of the message, consisting of sound, pressure, and tension, is indicated by the coupling constants of the deformed lipid membrane and ion channels. In this paper we define a message as an entity consisting of a chemical, light, sound, pressure, or tension that is received by a cell and changes its state.

### Message flow and message potential

In order to understand the time evolution of life phenomena based on physical constraints, we focused on the fact that biological systems are open systems that exchange messages, and worked on a method to represent life phenomena by message force fields.

**Figure 1:**
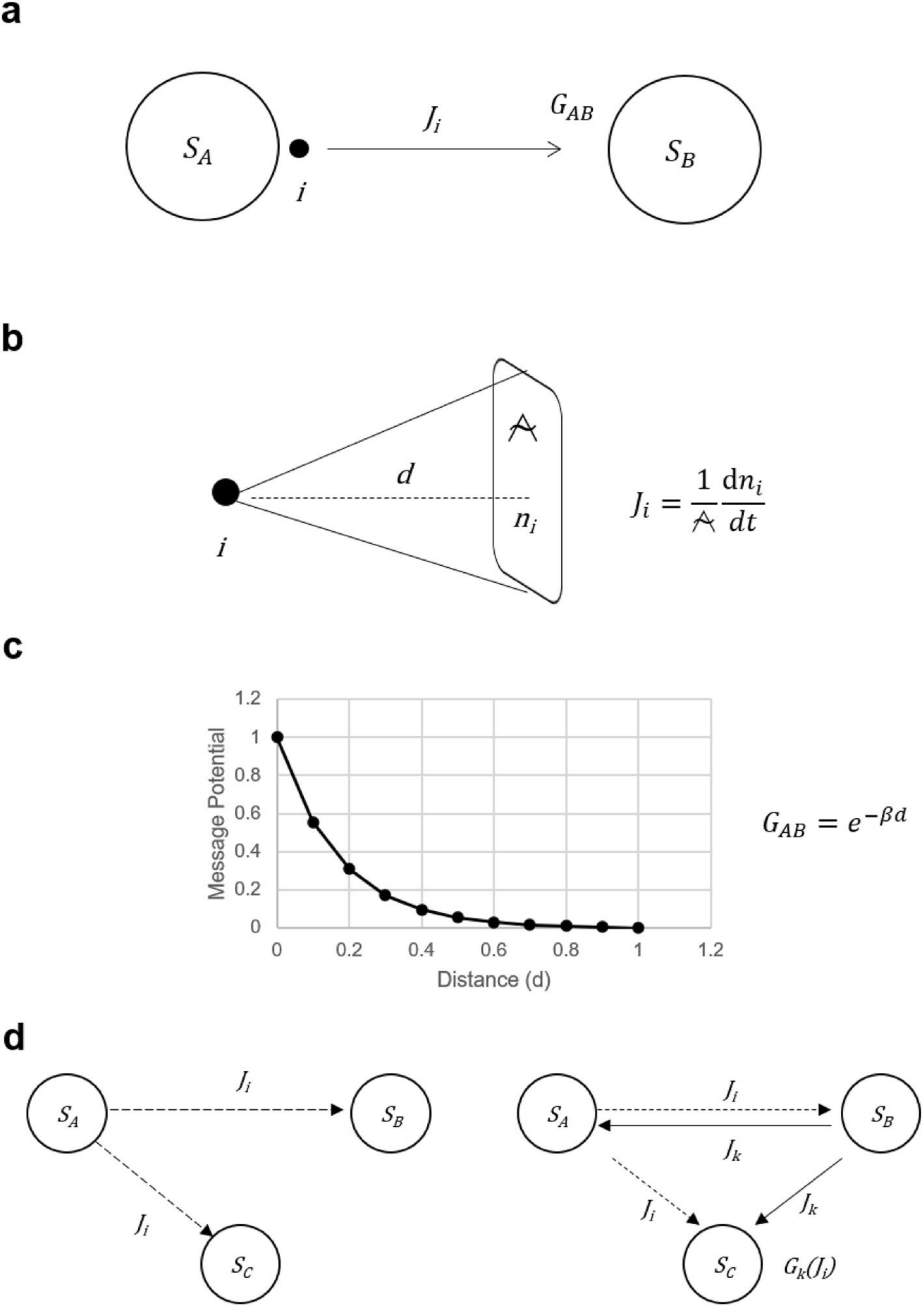
Message force field (**a**) The message *i* generated by system *S*_*A*_ spreads by diffusion, producing a diffuse flow *J*_*i*_. The system *S*_*B*_ receiving the message changes its state under the force of *G*_*AB*_ (**b**). The diffusion flow *J*_*i*_ is expressed as $\frac{1}{\mathcal{A}}\frac{{{\text{d}}n_{i} }}{{{\text{d}}t}}$, depending on the message quantity *n*_*i*_ passing through a unit area (⍲) in a certain direction in a unit time. (**c**) The force *G*_*AB*_ that changes the state of the system *S*_*B*_ receiving the message is exponentially dependent on the distance between the system *S*_*A*_ and the system *S*_*B*_. (**d**) Consider the case where system *S*_*B*_ and system *S*_*C*_ receive a message *i* generated by system *S*_*A*_ and a message *k* generated by system *S*_*B*_. In this case, the force *G*_*k*_ that changes the state of system *S*_*C*_ by message *k* is subject to the interference of *J*_*i*_.

Messages generated by living organisms commonly reach their counterparts by the principle of diffusion. When message *i* diffuses, a diffusion flow *J*_*i*_ is generated (Fig. 1a). This diffusion flow can be defined by the amount of message *n*_*i*_ that passes through a unit area (⍲) in a certain direction in a unit time as follows (Fig. 1b).

1 $$\begin{array}{*{20}c} {J_{i} = \frac{1}{\mathcal{A}}\frac{{{\text{d}}n_{i} }}{dt}} \\ \end{array}$$

Since messages have the function of changing the state of the system that receives them, a force field is created by the generation of the message. The force that drives the change depends on the concentration of the molecule in the case of chemicals. In contrast, in the case of light and sound messages, it depends on the intensity and spatio-temporal resolution that affect the identification of the sender. There is a threshold for the amount or intensity of a message that can change the state of a cell, since changing the state requires activation of the receptor. The force of the message that activates the receptor decays exponentially with distance. If the distance between the sender *(S*_*A*_*)* and receiver *(S*_*B*_*)* of the message is *d*, and the decay rate due to message diffusion is β, the force ${G}_{AB}$ to changes the state is expressed as follows (Fig. 1c).

2 $$\begin{array}{*{20}c} {G_{AB} = e^{ - \beta d} } \\ \end{array}$$

If the message is transmitted via cell adhesion molecules or tactile sensation, the message force occurs only when the distance between the message and the system is zero.

Identifying the state of a biological system based on measurements is essential for inferring the temporal evolution of the system based on the message force field. The state of a biological system can be identified by selecting and weighting features from a large number of measured features. In order to make inferences based on the message force field, it is necessary to know the set of states that the biological system can take. Usually, it is not possible to know the set of states from the data alone, since there are constraints on the sample used for analysis.

The set of states is understood as a probability distribution. If the probability density function of a state variable *x* is specified by a finite number of parameters "${\upxi } = \left( {{\upxi }_{1} , \ldots ,{\upxi }_{n} } \right)$", the entire distribution can be represented by a space $S = \left\{ {p\left( {x,{\upxi }} \right)} \right\}$ with *ξ* as the coordinate system. This is called an *n*-dimensional manifold^27^. A manifold is defined as a space in which each point *ξ* has an *n*-dimensional extent around it. A probability distribution family specified by a finite number of parameters is a finite-dimensional manifold. The geometric structure introduced here will be "invariant", reflecting the structure of the probability distribution family.

### Deriving the principle of time evolution from the flow and power of messages

To derive the principle of time evolution from the flow and force of messages, we introduced the concept of entropy. The entropy change of a system at time *t* can generally be expressed as follows^28,29^

3 $$\begin{array}{*{20}c} {\frac{\partial s}{{\partial t}} = \sigma - div \left( {j_{s} } \right)} \\ \end{array}$$

where *s* is the entropy density,* σ* is the entropy generation density, and *j*_*s*_ is the entropy flux density. From this equation, the entropy generation density *(σ*_*m*_*)* due to message flow is given by

4 $$\begin{array}{*{20}c} {{\upsigma }_{m} = \mathop \sum \limits_{i} G_{i} J_{i} } \\ \end{array}$$

where, *J*_*i*_ is the message flow and *G*_*i*_ is the force that changes the state of the system by the message *(i)*.

Since many messages interact in living organisms and ecosystems, the forces of state change driven by the messages interfere with each other (Fig. 1d). Taking this into account, the entropy generation density can be expressed as follows.

5 $$\begin{array}{*{20}c} {\sigma \left( {J_{i} } \right) = \mathop \sum \limits_{k} G_{k} \left( {J_{i} } \right)J_{k} } \\ \end{array}$$

Ziegler conducted a theoretical study to obtain *G*_*k*_* (J*_*k*_*)* explicitly and showed that the system formulated by (5) evolves in time to maximize entropy production^30–32^. Biological systems that evolve over time by exchanging messages maximize entropy production as they irreversibly transition from one non-equilibrium steady state to another.

### Pattern formation by symmetry generation and breaking

The maximum entropy generation principle is a general principle that constrains order formation in living organisms. Based on this principle, governing equations are introduced to explain specific biological phenomena.

#### Synchronization of systems interacting through messages

The primordial form of movement that occurs spontaneously in nature is periodic motion^15^. Where there is no periodicity, there is no time. Biological systems also exhibit periodicity, as exemplified by cell proliferation and differentiation, neuron firing, cardiomyocyte beating, immune system activity, brain waves, respiration, movement, and circadian rhythms^33,34^. There are two mechanisms for the formation of cell-scale autonomous oscillators. The first is based on the transcriptional network of genes, which occurs through negative feedback regulation of transcription factors^35^ The second is through reversible post-translational modifications of proteins^36^. Autonomous oscillations occur when the modification of a substrate fluctuates over time, depending on the combination of the enzyme catalyzing the chemical modification and the substrate receiving the modification. In the brain, autonomous oscillations are generated by central pattern generator circuits^37^.

We consider a biological system as a limit-cycle oscillator and represent it as an *n*-dimensional dynamical system as follows,

6 $$\begin{array}{*{20}c} {\frac{dX}{{dt}} = F\left( X \right)} \\ \end{array}$$

where *X* = *(X*_*1*_*, X*_*2*_*, ⋯, X*_*n*_*)* is an *n*-dimensional Euclidean space and *F* is a nonlinear real vector function of *X*. Biological systems are coupled by messages. By adding the force *G* of state change by message to the limit cycle oscillator, the life system can be represented as a coupled oscillator as follows,

7 $$\begin{array}{*{20}c} {\frac{dX}{{dt}} = F\left( X \right) + G\left( X \right)} \\ \end{array}$$

Individual multicellular organisms are formed from a population of cells, and ecosystems are formed from a population of individuals. Living systems are autonomous oscillators as well as composed of numerous elements that have the properties of autonomous oscillators. This property can be expressed as follows.

8 $$\begin{array}{*{20}c} {\frac{dX}{{dt}} = F_{j} \left( {X_{j} } \right) + \sum G_{jk} \left( {X_{j,} X_{k} } \right)} \\ \end{array}$$

The force that changes the state of the oscillator driven by the message delivered from oscillator *k* to oscillator *j* is denoted by ${G}_{jk}$.

**Figure 2:**
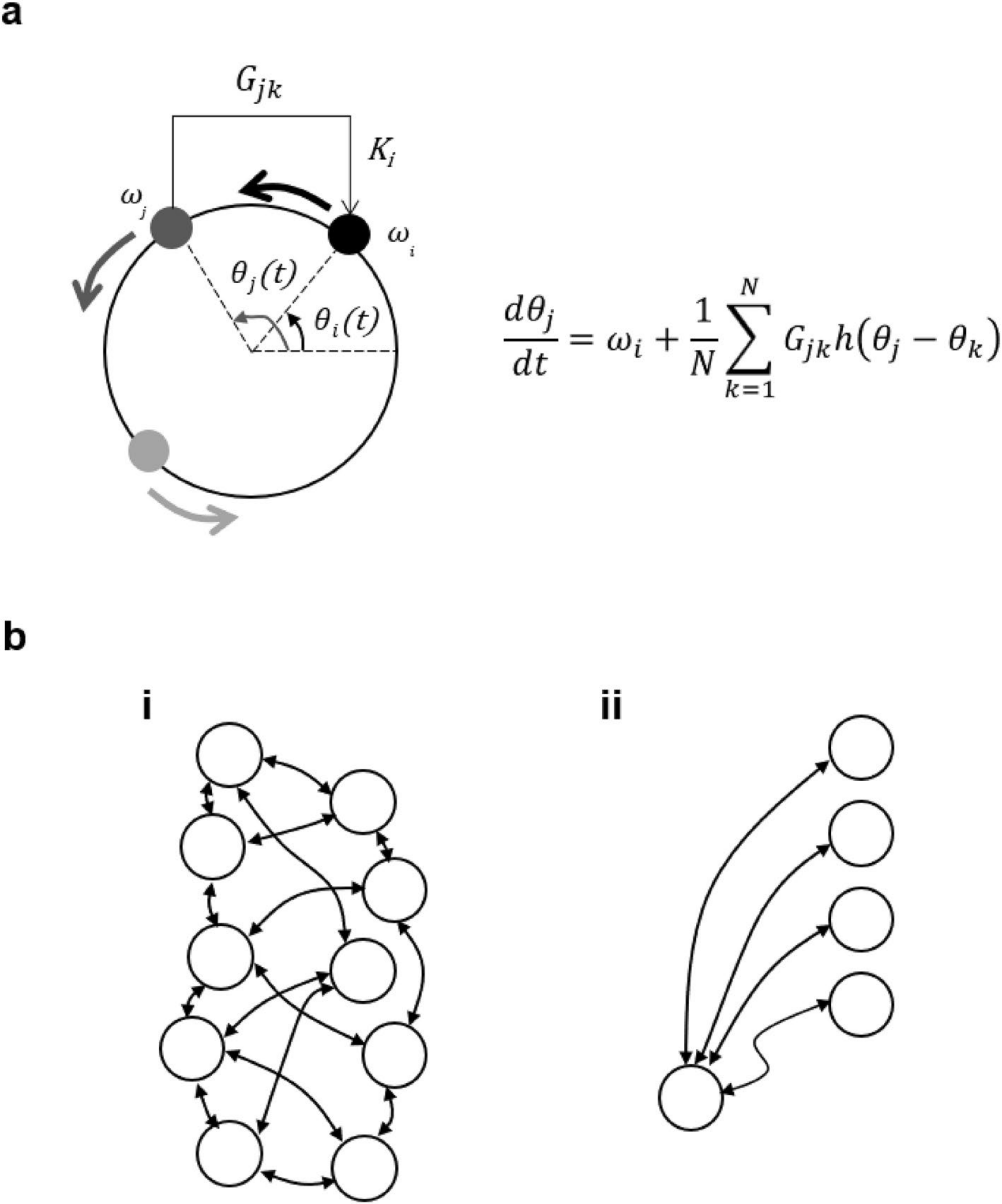
Synchronization of limit-cycle oscillators by message (**a**) If the natural frequency of oscillator *x* is $\omega_{x}$ and oscillators *j* and *k* are coupled by a *G*_*jk*_ force, the phase (θ) of oscillator *j* with *N* oscillators interacting by messages is shown as $\frac{{d\theta_{j} }}{dt} = \omega_{i} + \frac{1}{N}\mathop \sum \limits_{k = 1}^{N} G_{jk} h\left( {\theta_{j} - \theta_{k} } \right)$. (**b**) Biological systems form a variety of network structures. Here, we show a network structure in which elements are globally coupled and in which the network structure changes dynamically, as in the case of somite formation.

When limit-cycle oscillators are coupled bidirectionally by a certain coupling force *(G)*, synchronization between the oscillators occurs^19^. If the natural frequency of oscillator *x* is ${\omega }_{x}$ and oscillators *j* and *k* are coupled by a *G*_*jk*_ force, the phase of oscillator *j* when *N* oscillators are interacting by a message can be shown using the Kuramoto model^38^ as follows (Fig. 2a).

9 $$\begin{array}{*{20}c} {\frac{{d\theta_{j} }}{dt} = \omega_{j} + \frac{1}{N}\mathop \sum \limits_{k = 1}^{N} G_{jk} h\left( {\theta_{j} - \theta_{k} } \right)} \\ \end{array}$$

If the biological system is viewed as an oscillator capable of sending and receiving messages, synchronization causes irreversible transitions from one nonequilibrium steady state to another. In this case, entropy generation is maximized.

The state of the biological system is defined by which subsystems are synchronized and how. The nature of biological systems is characterized by the signaling networks formed between subsystems. Some biological systems have a global network structure in which each oscillator is equally coupled to other oscillators (Fig. 2b i), while others have a dynamically changing network structure, as in somitogenesis^39^ (Fig. 2b ii). Somitogenesis proceeds by a segmentation clock generated in the presomitic mesoderm (PSM). This converts to synchronization with the maternal circadian rhythm during functional maturation-stage^40^.

#### Breaking synchronization due to changes in the message force field

The temporal evolution of organisms is driven not only by the principle of steady state generation, but also by steady state breaking. Steady-state breaking is a universally observed phenomenon in living organisms and is a factor that generates biological diversity.

**Figure 3:**
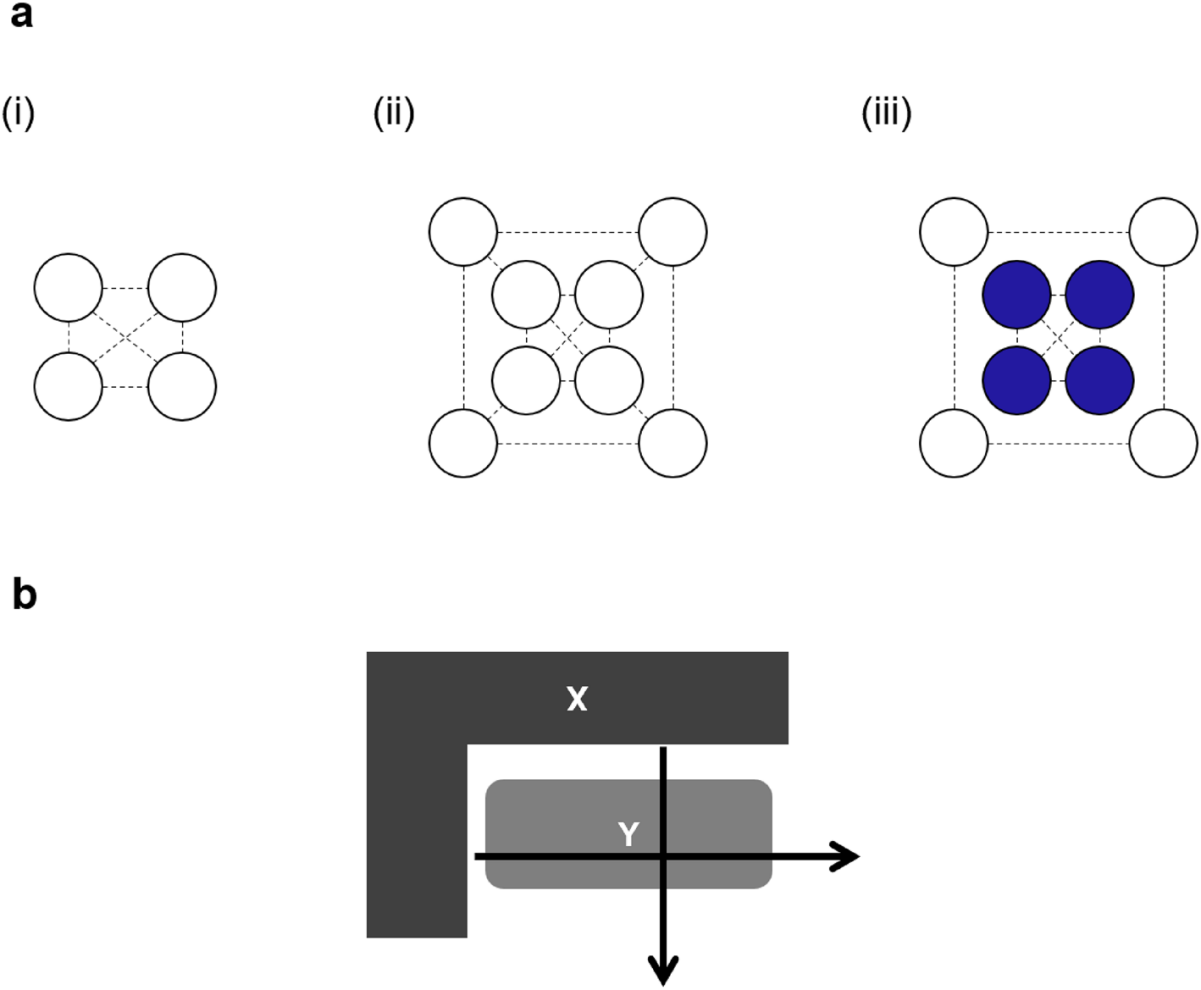
Spontaneous symmetry breaking due to cell proliferation and differentiation. (**a**) When cells in the same state come together to form a cluster, there is a difference in the message force field between the inside and outside of the cluster. In (i), all cells receive three streams of messages. In contrast, in (ii), the outer cells receive a flow of three messages, while the inner cells receive a flow of four messages. As a result, the cells inside and outside change to different states, and two populations of cells emerge. (**b**) When two different cell types proliferate to form two clumps X and Y, respectively, and are subsequently able to exchange messages with each other, a message force field is formed on different spatial axes, depending on the arrangement of the two clumps.

Consider the case of cells in the same state growing synchronously in a plane. If all cells produce an autocrine message, all cells are subject to the same message force when there are four cells, but when there are eight cells, there is a difference in message force between the center and the periphery (Fig. 3a i, ii). As a result, the overall synchronization is broken and separate synchronizations are generated in the central and outer cell populations (Fig. 3a iii). Now consider the case where two different cell types X and Y grow independently to form two clumps, which can then exchange messages. Depending on the arrangement of these two clumps, a force field of signaling is formed on different spatial axes (Fig. 3b).

Proliferation-related breaks in synchrony occur not only at the cellular level, but also at the individual level. When synchronized reproduction of predator and prey is broken, competition arises^41^. Predator–prey interactions occur not only through direct message exchange, but also through memory of messages to the environment. In termite population movements, indirect interactions through message memories stored on the field play an important role in pattern formation through self-organization^42^.

### Synthesis of different biological systems models

The multiple layers that make up a biological system interact through messages. Such a nonlinear interacting system cannot simply be simulated by the governing equations. Oscillatory phenomena do not appear unless the differential equations are nonlinear. Simulation by means of differential equations is not suitable for representing the behavior of concrete systems in which a large number of oscillators are intricately connected by messages. In contrast, machine learning has demonstrated the ability to create surrogate models of simulation that are accurate and fast. In surrogate models, the temporal evolution of a biological system is described by state transition probabilities. However, it cannot explain why biological systems exhibit certain changes. To overcome this problem, we considered integrating different time evolution models based on a discrete dynamics model. A discrete dynamical model represents the time evolution of a biological system as a transition from one steady state to another.

**Figure 4:**
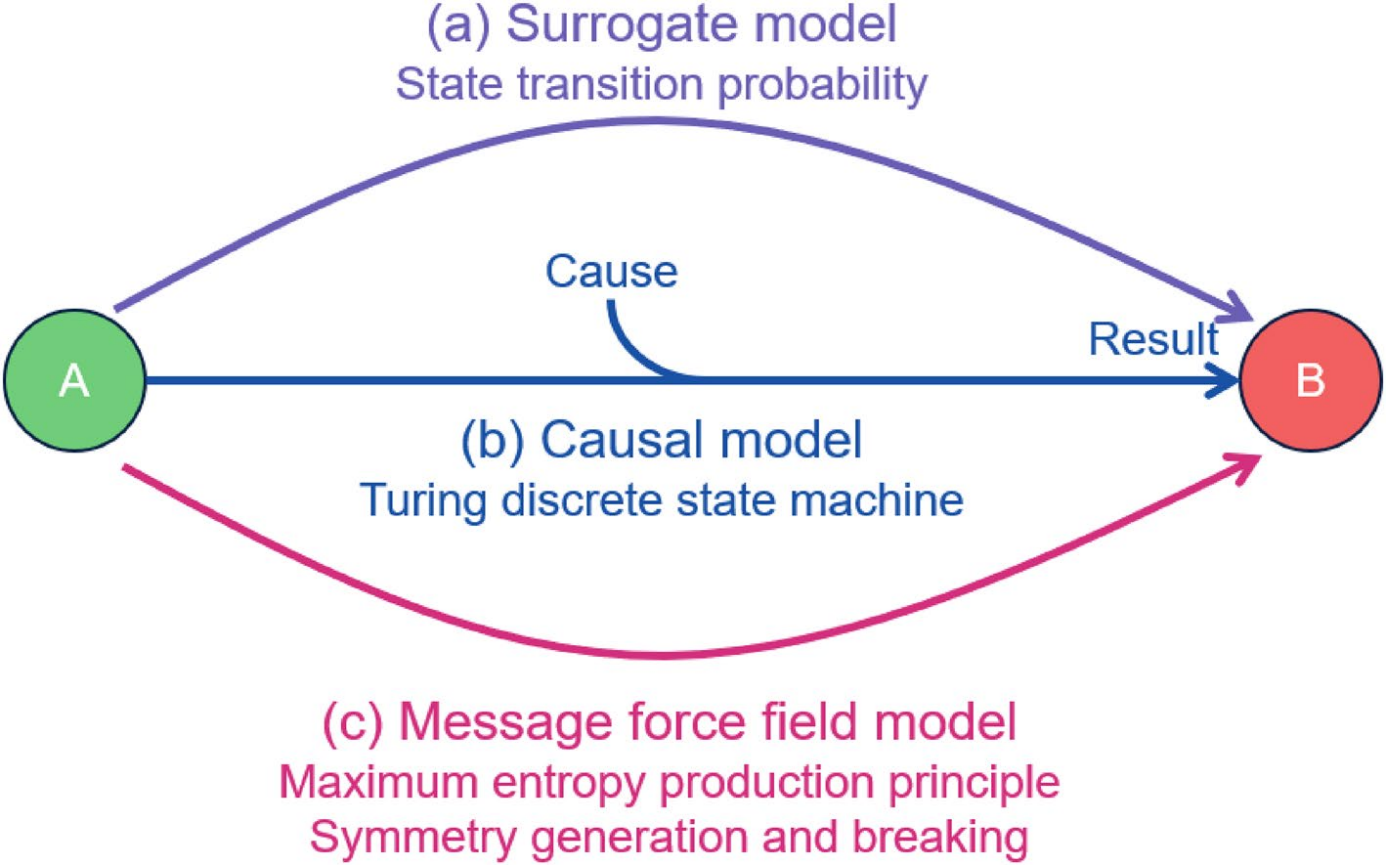
Isomorphism of different biological models The dynamic discrete model allows us to synthesize three different biological models. Consider the case of a biological system transitioning from state A to state B. In the surrogate model, the transition from state A to state B is represented by the state transition probability (**a**). In the causal model, on the other hand, the outcome of state B is considered to result from a certain cause for the precondition of state A (**b**). The message force field model explains that an irreversible change from state A to state B occurs within the constraints of the maximum entropy generation principle (**c**). The symmetry generated by the maximum entropy generation principle is broken by the change in the message force field.

In the causal model, the biological system is represented as changing from a steady state with one function to a steady state with another function due to a certain cause (Fig. 4a). This logic is generalized in Turing's discrete state machine model^43^. In the surrogate model, the transition from one steady state to another is expressed in terms of probabilities (Fig. 4b). In the case of the message force field, it is expressed in terms of a change in the message force field, which causes the system to transition to a new attractor state based on the principle of maximum entropy generation (Fig. 4c).

Thus, there is a similar structure in the time-evolving phenomena represented by the causal model, surrogate model, and message force field model. To synthesize the three models using this similarity, we introduced category theory. A category is a system consisting of an object and a morphism^44^.

In biology, the temporal development of organisms has been explained by the interaction of genetic and environmental factors. The category *(C)* of the causal model defines the object as the type of organism, and the morphism as the change in the organism's function due to genetic and environmental factors. If the elements of the finite set *M* are the functional changes induced by genetic and environmental factors, and the elements of the finite set *C* are the types, the function of the state transformation is shown by the following equation.

10 $$\begin{array}{*{20}c} {\mu :M \times C \to C} \\ \end{array}$$

The category *(D)* of the surrogate model defines the object as the state of the biological system identified by the feature vector and the morphism as the change in time. If the elements of the finite set* T* are points in time and the elements of the finite set *D* are states represented by feature vectors, the state transformation function is shown by the following equation.

11 $$\begin{array}{*{20}c} {\varphi :T \times D \to D} \\ \end{array}$$

The category *(E)* of the message force field model defines the object as the state of the biological system identified by the message network and the morphism as the state transformation induced by the message force field. If the elements of the finite set *Σ* are the message force fields and the elements of the finite set *E* are the states identified by the message network, the state transformation function is shown by the following equation.

12 $$\begin{array}{*{20}c} {\delta :\Sigma \times E \to E} \\ \end{array}$$

**Figure 5:**
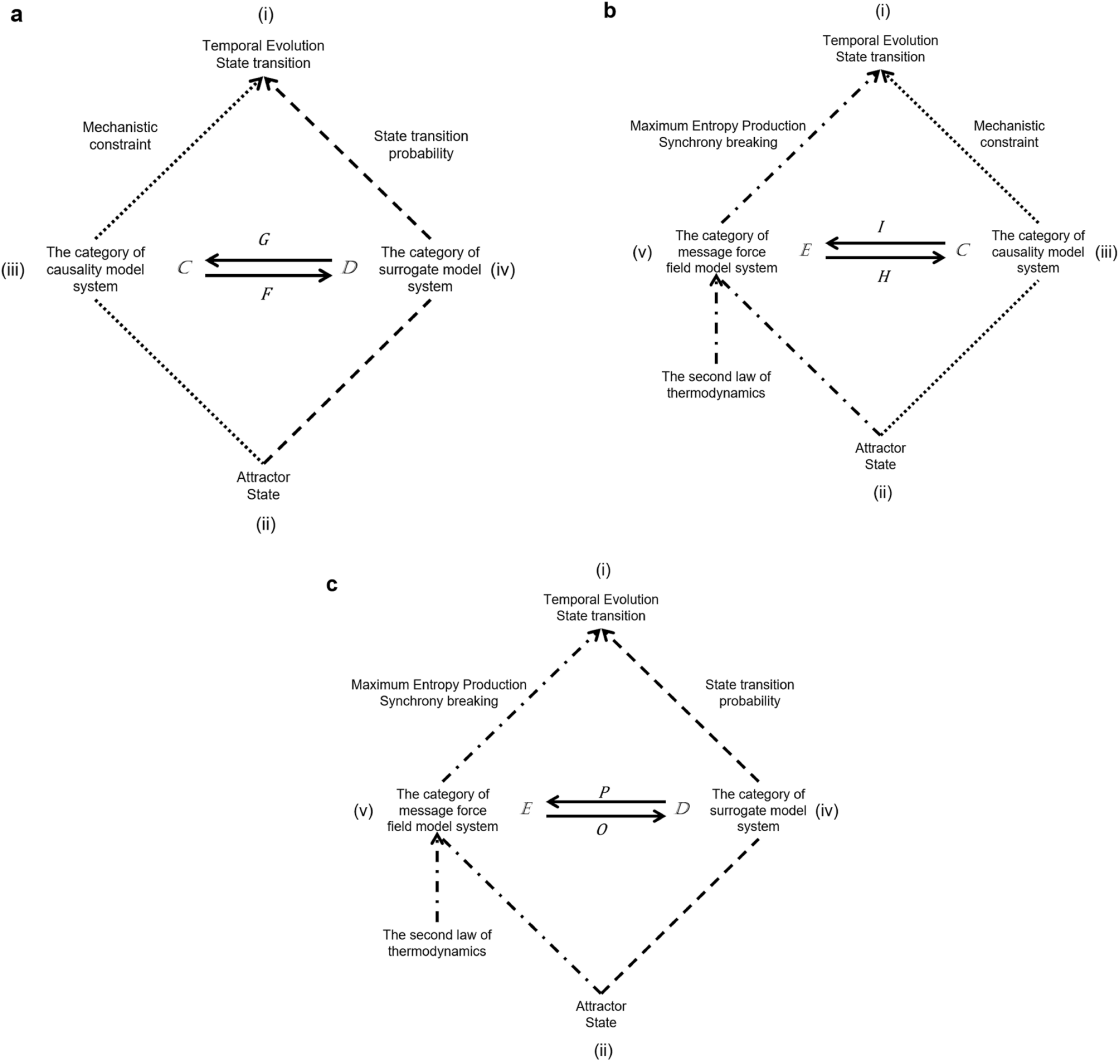
Synthesis of the model based on category theory Time evolution phenomena in biological systems are generally represented as attractor state transitions (i). To make this model versatile, it is necessary to standardize the identification of attractor states (ii). System identification is based on natural language-based types in the case of causal models (iii), feature vectors in the case of machine-learning-generated surrogate models (iv), and message networks in the case of message force field models (v). State transitions are represented by mechanistic constraints in the causal model (vi), by probabilities in the surrogate model (vii), and by the maximum entropy generation principle and synchrony breaking in the message force field (viii). To map the three models, we formulate them using the adjunction of category theory. The functor F from category (*C*) of the causal model to category (*D*) of the surrogate model and the functor G from category (*D*) of the surrogate model to category (*C*) of the causal model are adjoined. Similarly, bidirectional functor (I, H) from the category (*C*) of causal models and the category (*E*) of message force field models and bidirectional functor (O, P) from the category (*D*) of surrogate models and the category (*E*) of message force field models are adjoined.

The correspondence between categories can be made by functors. The case where there is a natural isomorphism between two functor is called an adjunction. In the three categories of the biological systems model, we can consider an adjunction between two categories, respectively (Fig. 5 and Methods).

### Application of governing equations to early embryonic development

There are no clearly visible pattern determinants (morphogens) in the blastocysts of mammalian embryos^45^. The blastocyst of a mammalian embryo differentiates into a trophectoderm and an inner cell mass. The trophectoderm gives rise to the placenta, and the inner cell mass gives rise to a new organism. Until the inner and outer cells are formed in the blastocyst, all cells have developmental plasticity and are identical. To explain how symmetry is broken in the blastocyst, we introduced a model of symmetry generation and breaking by message force fields introduced in this paper.

**Figure 6:**
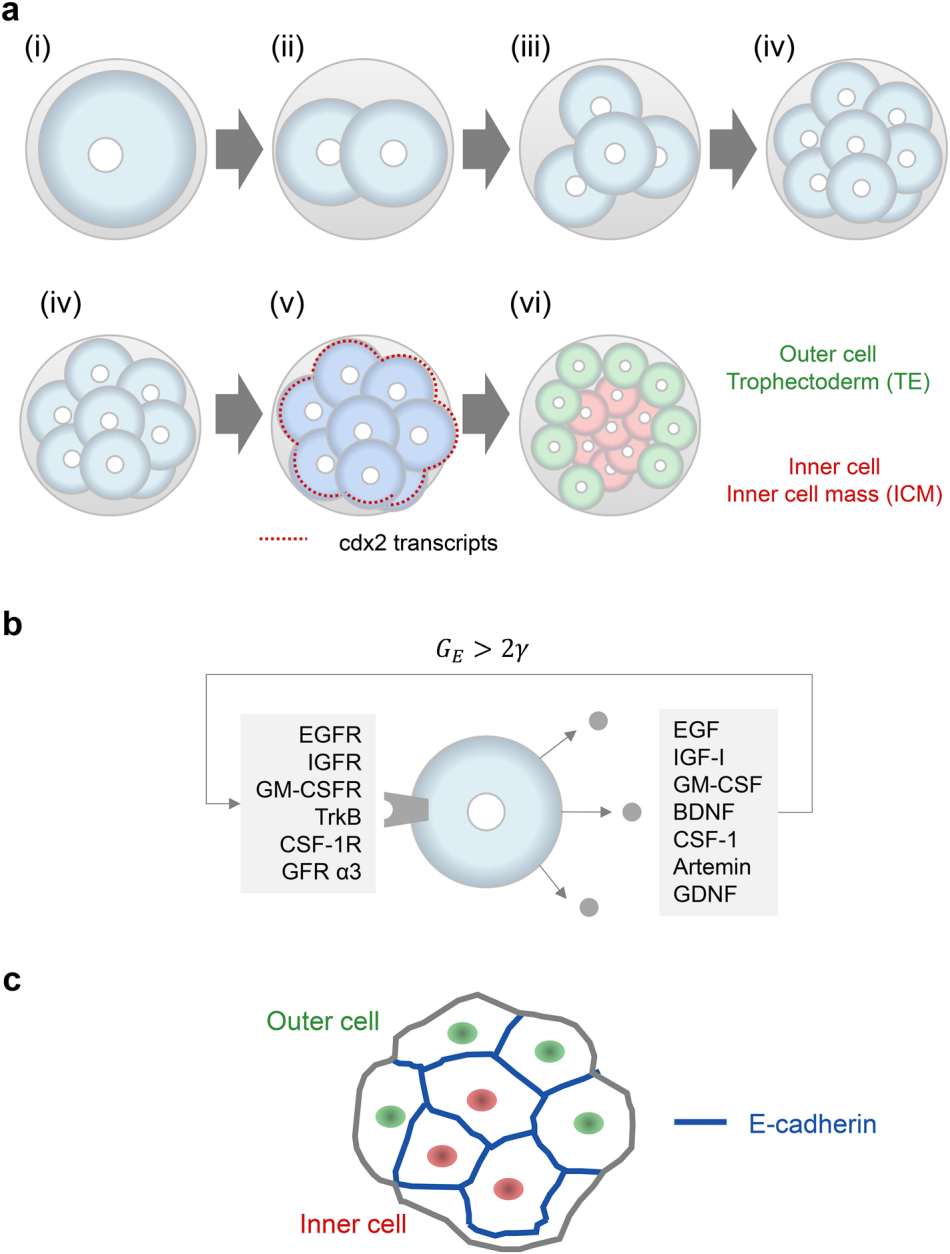
Inference of the blastocyst differentiation induction process by integrating the surrogate model and the message force field model. (**a**) The state changes of the embryo from fertilized egg to blastocyst are modeled based on the surrogate model as six different discrete states: fertilized egg (i), 2-cell stage (ii), 4-cell stage (iii), 8-cell stage (iv), compaction (v), and 16-cell stage (vi). (**b**) Cells from 2-cell stage to 8-cell stage express seven growth factors and six growth receptors. The synchronized cell division that occurs during this process is caused by the binding force expressed by the binding constants of the growth factors. (**c**) Compaction increases cell–cell adhesion and alters the force field of the message. The force field generated is asymmetric between inside and outside, resulting in the localization of cdx2 transcripts to the outside of the cell mass. As a result, an inner cell mass is induced in the interior and a trophic ectoderm is induced in the exterior. This process can be described as the breaking of synchrony and the reestablishment of synchrony.

The process from fertilized egg to blastocyst to differentiation into trophic ectoderm and inner cell mass can be represented by a six-stage discrete model: fertilized egg (S1), 2-cell stage (S2), 4-cell stage (S3), 8-cell stage (S4), compaction (S5), and 16-cell stage (S6) when differentiated into trophectoderm and inner cell mass (Fig. 6a).

Early embryonic development takes place in the oviduct and uterus without direct contact with reproductive tract tissues from fertilization to implantation. Cell proliferation in the early embryo is mediated by growth factors produced by the cells themselves. Cells in the embryo from the fertilized egg to the 8-cell stage produce seven different growth factors (messages): Epidermal growth factor (EGF), Insulin-like growth factor 1 (IGF-I), Granulocyte macrophage colony-stimulating factor (GM-CSF), Brain-derived neurotrophic factor (BDNF), Colony stimulating factor 1 (CSF-1), artemin, and Glial cell line-derived neurotrophic factor (GDNF), and six growth factor receptors: EGF receptor, IGF-I receptor, GM-CSF receptor, TrkB, CSF-1 receptor, and GFRα3^46^ (Fig. 6b). The embryonic cells form a uniform message force field for all cells by diffusing these autocrine factors.

To analyze this system based on Eq. (9), we introduce the following order parameter proposed by Steven Strogatz^47^.

13 $$\begin{array}{*{20}c} {R\left( t \right) = \left| {\frac{1}{N}\mathop \sum \limits_{j} e^{{i\theta_{j} \left( t \right)}} } \right|\Theta \left( t \right) } \\ \end{array}$$

In a synchronous state, *R*(*t*) will be close to 1, while *R*(*t*) will be close to 0 if the phases are disjoint. Here, $\frac{1}{N}{\sum }_{j}{e}^{i{\theta }_{j}}=R(t){e}^{i\Theta }$ for the order parameter.

Suppose the natural frequency ω in Eq. (9) follows a probability distribution *f(ω)*. Let us assume that *f(ω)* follows the Cauchy distribution, which is shown below.

14 $$\begin{array}{*{20}c} {f\left( \omega \right) = \frac{1}{\pi }\frac{\gamma }{{\left( {\omega - \omega_{0} } \right)^{2} + \gamma^{2} }}} \\ \end{array}$$

where ${\omega }_{0}$ is the center of the distribution and *γ* is the spread of the distribution.

The value of the order variable (${R}_{\infty }$) at equilibrium (*R*(*t*)) can be expressed from the binding strength ${G}_{E}$, indicated by the binding constants to growth factors and growth factor receptors, and the spread of the distribution *γ* of the natural frequencies of individual cells as follows

15 $$\begin{array}{*{20}c} {R_{\infty } {}\sqrt {1 - \frac{2\gamma }{{G_{E} }}} } \\ \end{array}$$

This equation holds only when ${G}_{E}>2\gamma$; when ${G}_{E}<2\gamma$, ${R}_{\infty }=0$. In other words, if the variation in natural frequencies between cells is large, they will not synchronize unless there is a sufficiently large coupling strength. The synchronization of cell division from the fertilized egg to the 8-cell stage suggests that the variation in natural frequencies between cells is small and the concentration of growth factors is sufficiently high. This is consistent with what is observed in embryos^48^.

At the 8-cell stage, cell adhesion is strengthened and a state called compaction occurs (Fig. 6c). E-cadherin is involved in increasing cell adhesion^49^. E-cadherin molecules bind to each other (homophilic binding) and form an adhesive bond between opposing cell membranes. This homophilic bond creates a new message force field. This message force field breaks the previous synchronization. In the center of the mass, there is more contact between the cell membranes, while outside the mass, the contact is less. This means that in the center, the E-cadherin signal is stronger and the concentration of message produced by the cell is higher.

Breaking the symmetry of the message force field causes cdx2 mRNA to localize to the outer cells^50,51^. Differences in the pattern of gene expression between the outer and inner cells result in cell differentiation. The outer and inner cells form separate synchronizations, forming two cell populations. These are the trophectoderm and the inner cell mass.

Thus, the surrogate model and the message force field model can explain phenomena that are difficult to explain with conventional causal models and governing equation simulations.

## Discussion

In order to describe life phenomena based on physics-based constraints, we asked the following questions: What is the "driving force" that causes spontaneous biological change? Why do biological changes occur, stop at a certain point, and start again once they have stopped? In this paper, we address this question by introducing a model that describes living systems in terms of limit-cycle oscillators that send and receive messages. First, we introduced the theory of message force fields because messages have a driving force that changes the state of the system that receives them. We show that when a living system undergoes irreversible change under this message force field, subsystems synchronize through messages to minimize message potential and maximize entropy. Next, we showed that the steady state of the system generated by synchronization is broken by the proliferation of subsystems, generating new message potentials. This potential induces a new synchronous state. The synchronization resulting from message potentials and the breaking of synchronization resulting from system proliferation explain the properties of heterogeneity, hierarchy, self-organization, adaptability, memory, nonlinearity, and uncertainty common to life phenomena.

Macroscopic changes observed in evolution, such as the differentiation of new species, have been explained by an algorithm of genetic variation and natural selection^7^. In contrast, Kaufman explained evolution from self-organization using an algorithm called the random Boolean network, an extension of the cellular automaton^52^. Organisms have evolved from unicellular to multicellular organisms, and from animals with simple brains to humans with advanced brains. The observables and principles that determine the direction of such evolution are placed outside the discussion in the theory of natural selection. The self-organization model was meant to complement this problem. However, the self-organizing model eliminated the natural selection theory.

The model proposed in this paper, in which the biological system is regarded as an oscillator, makes it possible to integrate the natural selection theory and the self-organization model into one. Self-organization through synchronization leads to the emergence of adaptive traits. On the other hand, when an individual organism loses synchrony with its environment, natural selection occurs through competition until a new synchrony is generated.

The exchange of diverse messages between systems occurred, causing the organism to evolve toward a complex, multiscale structure. Approximately 3.8 billion years ago, the common ancestor of all living things was born and divided into two types of prokaryotes, Archaea and Bacteria. Billions of years later, the two fused into a single cell, giving birth to eukaryotes^53^ Bacteria fused into Archaea later became mitochondria. The fusion of anaerobic Archaea with bacteria capable of oxygen respiration and detoxification was necessary for them to survive in an environment with elevated oxygen levels. This symbiosis is made possible by the exchange of messages between cells and mitochondria. Mitochondria exchange messages with cells by accepting proteins synthesized in the cytoplasm via the Tom20 protein on their outer membrane^54^.

Similarly, in the evolution of multicellular organisms from unicellular organisms, the exchange of messages between cells was essential. In long-term evolutionary experiments in which yeast cells were made multicellular by adhesion, no exchange of messages occurred between yeast cells^55^. Yeast cells grow through division and have a branched, tree-like structure. This occurred to coordinate competing energies and resources during the self-organization process. The morphology generated from a cluster of cells that exchange messages is very different from the multicellularity of yeast, which does not exchange messages.

Human cognition results from the integration of multiple senses consisting of exteroception, proprioception and interoception^56^. Each sensory area of the brain contains neurons that are active in response to specific sensory signals and a coordinate system that represents variables in the external world. However, it is not clear how multisensory integration occurs. This is called the brain binding problem^57^. Neuroscience has two views of cognition^58^. The "outside-in" view sees perception as the correct representation of sensory signals from the outside world^59^. The "inside-outside" view considers perception to be the localization of sensation to things in the world that give rise to sensation^60^. This is called projection. A projection is a reality constructed by the brain.

In the artificial intelligence domain, the binding problem was solved by a method of series transformation that relies on an attention mechanism called transformer^61^. In the transformer, the spatio-temporal structure of the object is taken apart and represented by a vector, and then the information of the spatio-temporal structure is encoded and added to the vector. In artificial neural networks, the error back-propagation algorithm is used to encode the spatio-temporal structure.

Two biological back-propagations, the temporal error model and the explicit error model, have been proposed to incorporate the results of artificial intelligence into models of the brain^62^. Within the "outside-in" view, learning is done to seek the correct output. Learning, assuming the correct answer, can be modeled as a process of convergence to an equilibrium state by the energy minimization principle. As the network reaches equilibrium, the weights are modified. The time error model minimizes the Hopfield energy, while the explicit error model minimizes the free energy.

The brain is a non-equilibrium open system, generated by self-organization and spontaneously active^58^. In the "inside-outside" view, it is necessary to introduce the principle of entropy generation to describe the dynamism of the brain. Neurons or neural circuits are spontaneously active as nonlinear oscillators, and interconnected neurons or neural circuits move toward synchronization by the principle of maximum entropy generation. There is plasticity in the way neurons are connected and the intensity of connections. As a result, the brain has a potentially vast repertoire of spontaneous neural activity. From an “inside-out” perspective, learning can be modeled as the process of matching spontaneous neural activity in the brain with external events captured through the sensory organs. The concept of a message force field presented in this paper can be introduced into this modeling.

There is an isomorphism between the "outside-in" and the "inside-out" views of cognition: representation or projection through multisensory integration. These two views can be synthesized by means of the adjoint functor presented in this paper.

Surrogate models generated by machine learning from the data were found to be able to represent various natural phenomena with high accuracy. However, living organisms are multi-hierarchically connected non-equilibrium and non-linear open systems, and have an uncertain nature represented by scale interferences and critical phenomena. Scale interference refers to the influence of the behavior of microscopic elements by macroscopic states, which in principle cannot be predicted from microscopic to macroscopic. At the critical point, the system is not smooth and averages do not behave well, which violates the assumptions of standard calculus and statistics. This implies that both causal models, which predict the macro from the micro, and surrogate models, which express probabilities by learning which state comes next given a state, are condition-dependent.

Representing irreversible processes using entropy generation and dissipative functions is useful in general terms, but it is not useful for describing individual, specific life phenomena. The message force field theory presented in this paper can overcome this challenge. This theory allows us to obtain specific information about the flow of messages and the force of state change. We hope that this theory will pioneer a new biomedical science by integrating information science, biology, and physics.

## Methods

### Categories of causal models *(C)*

The steady state of the system is represented by the types and their biological-functions. Functional changes in the system are induced by genetic or environmental factors. The category of this system can be formulated as follows.

1. *μ* is a non-empty finite set whose elements are changes in biological function induced by genetic and environmental factors,
2. *C* is a non-empty finite set whose elements are the type and biological function of the system,
3. function *μ : M* × *C → C* denotes the change in biological-function induced by genetic and environmental factors.

### A category of surrogate model systems *(D)*

In the surrogate model, identification of system states is based on feature selection and weighting. Features that are similar to the output are weighted and redundant features that show similar changes are removed. The set of states occupies a subspace of the state space. Changes from one state to another are represented by probabilities. The category of this system can be formulated as follows.

1. The set of states *D* does not occupy the entire state space, but is distributed among geometric subspaces such as manifolds; *D* is a subspace of R^n^ for some *n ∈ N*, with the usual topology inherited from the Euclidean distance,
2. T is a finite non-empty set, called points in time. The time space is the set of natural numbers *N* and has a monoidal structure < *N:* + *, 0* > ,
3. function *φ*: *T* × *D* → *D* denotes the time evolution of the system.

### A category of message force field model *(E)*

In the message force field model, the characteristics of the system are defined by the network structure in which messages are sent and received. Changes in the network structure are induced by message force field. The category of this system can be formulated as follows.

1. Σ is a finite non-empty set, called the message force field,
2. *E* is a non-empty finite set whose elements are network structures,
3. Function *δ: Σ* × *E → E* denotes a transformation of the network structure.

### An adjunction between the category of causality model (*C*) and the category of surrogate model (*D*)

An adjunction between the category of causality model (*C*) and the category of surrogate model (*D*) is a pair of functors

$$F:D \to C\, {\text{and}}\,G:C \to D$$

Together with a natural isomorphism whose component for any objects is:

$$C\left( {c \, \leftarrow \, G\left( d \right)} \right) \simeq D\left( {F\left( c \right) \, \leftarrow \, d} \right).$$

### An adjunction between the category of message force field model *(E) *and the category of causality model *(C)*

An adjunction between the category of message force field model (*E*) and the category of causality model (*C*) is a pair of functors

$$H:C \to E\,{\text{and}}\,I:E \to C$$

together with a natural isomorphism whose component for any objects is:

$$E\left( {e \, \leftarrow \, I\left( c \right)} \right) \simeq C\left( {H\left( e \right) \, \leftarrow \, c} \right).$$

### An adjunction between the category of message force field model *(E) *and the category of surrogate model *(D)*

An adjunction between the category of message force field model (*E*) and the category of surrogate model (*D*) is a pair of functors

$$O:E \to D\,{\text{and}}\,P:D \to E$$

together with a natural isomorphism whose component for any objects is:

$$E\left( {e \, \leftarrow \, P\left( d \right)} \right) \simeq D\left( {O\left( e \right) \, \leftarrow \, d} \right).$$

## Data Availability

All data generated or analyzed during this study are included in this published article.
